# Three Unrelated Children With Childhood Apraxia of Speech: Exome Sequencing and Functional Gene Analysis Imply a Role of Laminin‐511 in Early Neurodevelopment

**DOI:** 10.1155/crig/9927839

**Published:** 2026-02-13

**Authors:** Caitlin Raaz, Laurel Bruce, Madhavi Ganapathiraju, Judith Klein-Seetharaman, Li Liu, Valentin Dinu, Marjan Chapi, Eunhyo Kim, Yookyung Kim, Tiffanie White, Beate Peter

**Affiliations:** ^1^ Department of Communication Sciences and Disorders, University of Northern Colorado, Greeley, 80639, Colorado, USA, unco.edu; ^2^ College of Health Solutions, Arizona State University, Phoenix, 85004, Arizona, USA, asu.edu; ^3^ Department of Biomedical Informatics, University of Pittsburgh, Pittsburgh, 15260, Pennsylvania, USA, pitt.edu; ^4^ College of Veterinary Medicine, University of Georgia, Athens, 30602, Georgia, USA, uga.edu

**Keywords:** cerebellum, childhood apraxia of speech, fine and gross motor delay, gene expression in the prenatal brain, protein–protein interaction, whole exome sequencing

## Abstract

Childhood apraxia of speech (CAS) is characterized by motor discoordination in the speech domain and also in fine and gross motor systems, implicating the early developing cerebellum. Comorbidity with autism spectrum disorder (ASD) and other neurodevelopmental conditions has been observed. The genetic etiology is highly heterogeneous. Here, we present three unrelated individuals with CAS and concomitant fine and gross motor involvement but different genetic variants of interest. The DNA of the cases and their parents underwent exome sequencing and variant filtering. Using publicly available data, the genes of interest derived from the variants were investigated for expression rates in the early developing brain. Known and putative protein–protein interactions among the genes of highest confidence were identified. Of 28 variants in 28 different genes, variants with highest confidence were situated in *FOXN4*, *LAMA5*, *LAMB1*, *LRRK2*, and *USP17L2*. High gene expression rates in the developing cerebellum were observed for *LAMA5* and *LAMB1*. These genes encode the α5 and β1 subunits, respectively, of the heterotrimeric extracellular laminin‐511 complex, a major component of the basal membrane in many tissues. Network analysis of the five high‐confidence genes required expansion with only one additional gene, *CDK6*, to arrive at a fully connected network. The addition of four genes and inclusion of transcriptional regulation as an additional edge type allowed connecting all 28 genes of interest to arrive at a dense connectome with 32 nodes and 73 edges, representing a network enrichment with *p* value of < 0.001, suggesting that our network has significantly more interactions than expected under random conditions. We conclude that high levels of genetic heterogeneity converge on a functional gene network governed by stimulation of cells through laminin‐511 with shared direct or regulatory expression in the developing cerebellum and phenotypic overlaps of CAS, ASD, and other neurodevelopmental disorders.

## 1. Introduction

Among the various forms of childhood speech sound disorders, childhood apraxia of speech (CAS) is a severe form that can substantially impair communication abilities. It is a motor speech disorder at the level of motor planning/programming and coordination. The American Speech–Language–Hearing Association (ASHA) defines CAS as “a neurological childhood (pediatric) speech sound disorder in which the precision and consistency of movements underlying speech are impaired in the absence of neuromuscular deficits” [1]. The speech of children with CAS is characterized by inconsistent speech sound errors, vowel distortions, lack of differentiation between stressed and unstressed syllables, mis‐stressed syllables, difficulty with multisyllabic words [2], slow diadochokinetic (DDK) rates during production of strings of meaningless syllables, e.g., as “papapa …,” “tatata …” [3–5], and unusual pauses disrupting speech production [6–9].

Beyond the characteristic speech traits, CAS is commonly comorbid with a range of other signs and symptoms. We and others have shown that for children with CAS, motor processes that require complex coordination are affected more than those requiring repetitive or simple movements, not only during DDK production of di‐ and trisyllables (e.g., “patapata …,” “takataka …,” “patakapataka …”) but also during complex fine and gross motor tasks [3, 4, 10–17]. This is likely due to biological mechanisms that affect complex motor coordination generally. Other conditions that are frequently seen in children with CAS include history of feeding difficulties, developmental coordination disorder, disordered receptive and especially expressive language development, deficits in reading and spelling, intellectual delays, anxiety, depression, hypotonia, and dysmorphic features [11, 13, 18, 19]. Given the cerebellum’s role in motor coordination, temporal integration, and sequencing of information across domains including spoken and written language [20–22], we hypothesize that the cerebellum is one locus of impairment for CAS.

There is growing evidence that CAS is influenced by genetic variations, but the underlying mechanisms are not yet well understood. As outlined in various reviews [23–25], the genetic etiology appears to be highly ∗heterogeneous^1^ in that different genes have been implicated in different individuals with CAS. In addition, various types of disruptions have been observed, including ∗single nucleotide variants (SNVs) and ∗copy‐number variations (CNVs). CAS can be inherited or ∗de novo. For instance, in one extended family with CAS, an inherited SNV in the *FOXP2* gene was identified [26–28]. In a small number of additional inherited and *de novo* cases, *FOXP2* was found to be disrupted by SNVs, CNVs, or ∗translocations [23]. Deletions in the ∗16p11.2 band were seen in several unrelated cases with CAS [29–32]. Additional genes implicated in CAS include *ANKRD12*, *ATP13A4*, *CDH18*, *CHD3*, *CNTNAP1*, *CNTNAP2*, *ELKS*, *FLCN*, *FOXP1*, *KAT6A*, *KIAA0319*, *MYO10*, *NCOR1*, *NEK8*, *NIPBL*, *SETD1A*, *SMCR8*, *SNF385D*, *TNRC6B*, *WDR5*, and *ZFHX4* [24]. Apraxic speech is also seen in the presence of various syndromes of genetic etiology, for instance, classic galactosemia (CG) resulting from disruptions in the *GALT* gene [33, 34], *BCL11A*‐related Intellectual Development Disorder with Persistence of Fetal Hemoglobin syndrome (BCL11A‐IDD), also referred to as Dias–Logan syndrome (OMIM #617101) [35–39], Rolandic epilepsy [40], autism spectrum disorder (ASD) [41–44], and Bainbridge–Ropers syndrome [45]. According to a recent review [46], motor speech disorders are common among individuals with neurodevelopmental disorders caused by variants in genes that regulate gene expression via chromatin remodeling. The authors suggest that chromatin architecture and epigenetic mechanisms have specific effects on the brain’s speech and language networks.

Given the high levels of genetic heterogeneity in CAS, a key hypothesis (Hypothesis 1, H1) posited here is that diverse genetic variations have a converging influence on brain structures and functions and, downstream from there, on the observable clinical ∗phenotype, a many‐to‐one (gene to brain structure) relationship. A second hypothesis (H2) is that where certain brain structures are implicated, disruptions in genes expressed in these brain structures can produce diverse phenotypes in the same individuals as ∗pleiotropic effects due to multiple functions controlled by the brain structure, a one‐to‐many relationship (brain structure to traits). Thirdly, genetic changes that produce neurodevelopmental traits, such as CAS, are thought to occur in genes that are expressed in the earliest developing brain (H3). Fourth, as mentioned, one brain structure of particular interest for CAS and several associated traits, such as motor discoordination in the speech, fine, and gross motor system, and balance difficulties, is the cerebellum [12, 14, 45] (H4). A fifth hypothesis (H5) is that various genes that are implicated in CAS are functionally related.

The following three studies illustrate these hypotheses. First, in a study of 19 individuals with CAS [18], the eight implicated genes, all checked for expression in the prenatal developing brain, were *CHD3*, *KAT6A*, *MKL2*, *SETD1A*, *SETBP1*, *TNRC6B*, *WDR5*, and *ZFHX4*. These genes are functionally associated in that they interact with each other or other genes known to influence speech ability during embryonic development. Second, in 11 of 34 probands with CAS, 10 genes were identified, *CDK13*, *EBF3*, *GNAO1*, *GNB1*, *DDX3X*, *MEIS2*, *POGZ*, *SETBP1*, *UPF2*, and *ZNF142* [11]. With the exception of *GNAO1* and *GNB1*, which are ∗G protein–related genes, all of these genes are involved in ∗DNA binding [11]. Third, in a study of 70 unrelated individuals with CAS, high‐confidence variants were found in 18 genes (*ARHGEF9*, *BRPF1*, *DDX3X*, *DIP2C*, *ERF*, *HRNPNK*, *KDM5C*, *PHF21A*, *PURA*, *RBFOX3*, *SETBP1*, *SETD1A*, *SETD1B*, *SHANK3*, *SPAST*, *TAOK2*, *TRIP12*, and *ZBTB18*) [13]. Note that *SETBP1* was found in all three studies, and *SETD1A* and *DDX3X* were each listed in two of the studies. The fact that of the 32 genes implicated in the three studies combined, only three were found in more than one individual is consistent with high levels of heterogeneity (H1).

The first of these three studies [18] listed fine or gross motor impairment in 16 of the 19 participants (84%). The second study [11] listed medical and neurodevelopmental conditions for all 34 participants, and the third [13] listed these only for the 18 participants with pathogenic or likely pathogenic variants. In these latter two studies, respectively, gross and fine motor delays were present in the largest number of participants (73% and 89%, respectively), followed by dysmorphic features (36%, 61%), problems with attention (21%, 22%), signs of ASD (18%, 11%), and conditions related to the immune system (9%, 17%). This diverse assortment of signs and symptoms is consistent with pleiotropy (H2).

In all three studies, the genes associated with CAS were co‐expressed in the prenatal and early postnatal brain (H3). Gene set enrichment analysis based on 15 genes from all three studies that were highly co‐expressed showed that this gene set included a high proportion of genes involved in ∗chromatin organization and ∗transcriptional regulation, consistent with functional networks among implicated genes (H5) [13].

With respect to the comorbidity of CAS and ASD, it is noteworthy that of the 32 combined genes implicated in the Eising et al. [18], Hildebrand et al. [11], and Kaspi et al. [13] studies, 24 are listed in the Simons Foundation Autism Research Initiative (SFARI) database (https://www.sfari.org, Version October 2025, 1255 genes) [47], as relevant for ASD (Fisher’s exact *p* < 2.2*e* − 16). Of these, 13 are included among the 240 genes classified as having the highest level of evidence (Fisher’s exact test *p* value < 2.2*e* − 16). Further support for a shared genetic etiology in CAS and ASD is a 16p11.2 microdeletion region. Of 11 children with ASD who had deletions in this region, two were nonverbal and all others had disordered speech consistent with CAS [29], whereas in a group of 136 16p11.2 deletion carriers with ASD, 79% had speech articulation difficulties [43]. Consistent with our hypothesized gene–cerebellum–phenotype cascade, the cerebellum has been implicated as a locus of impairment in ASD [48]. Thus, we posit that disruptions in various genes, all expressed in the early developing cerebellum, can produce traits seen in individuals with CAS, ASD, or both. The high comorbidity with gross and fine motor delays [3, 4, 10–17] also supports this hypothesis (H4).

From the foregoing, it is clear that the genetic etiologies of CAS are complex and highly heterogeneous. Additional studies are needed to discover novel CAS genes, add insights to functional networks, and contribute to the knowledge base of genotype–phenotype patterns.

Biological domain knowledge, such as biological pathway information or protein–protein interactions (PPIs), can supplement statistical and data mining approaches to identify biomarkers associated with disease [49, 50]. These approaches are particularly useful in the study of complex disorders that could be influenced by the interaction of multiple genes and environmental factors. We have developed methods for incorporating domain knowledge in the analysis of association between genomic variants and disease [49], and we have applied these methods to study the etiology of various disorders [41, 43, 44, 51–61]. Given our previous work in PPI prediction, we had created a high‐confidence human ∗interactome model that has been extensively validated experimentally [62] and that only considers those interactions for which the confidence of prediction is very high [62–72]. We apply these analytical approaches to explore the association between genetic variants and CAS.

Here, we present three unrelated cases with CAS and associated cerebellar motor signs, whose DNA samples underwent ∗whole exome sequencing (WES). In addition to ∗exome ∗variant analysis, expression and co‐expression patterns in the early developing brain, PPI networks, and genetic overlaps with other neurodevelopmental conditions are addressed. To our knowledge, this study is the first to interrogate not only DNA variants in cases with CAS but also functional gene networks, PPIs, and gene expression patterns in the early developing brain and specifically the cerebellum. Our goal was to contribute evidence to the hypotheses mentioned above: H1 (heterogeneity): Different genes influence similar phenotypes via expression in the same brain regions, explaining why individuals with similar CAS phenotypic profiles may have variants in different genes; H2 (pleiotropy): Different genes converge on brain structures that govern multiple phenotypes in individuals with CAS; H3 (prenatal gene expression, neurogenesis, and neural architecture): To produce a neurodevelopmental phenotype, such as CAS, causal genes are expressed in the earliest developing brain; H4 (cerebellar locus of impairment): Given the phenotypic traits of motor discoordination in the speech, fine, and gross motor system in CAS and the phenotypic overlaps with ASD, the early developing cerebellum is a likely locus of impairment; and H5 (functional networks, gene–gene interactions, and PPIs): Different genes implicated in CAS are part of functional networks that influence neurodevelopment in characteristic ways.

We believe that our findings may have important translational impact by providing interprofessional insights to members of the genetics/genomics and clinical linguistics communities. Speech‐language pathologists are among the key professionals diagnosing and treating individuals with CAS. Aware of the rapidly increasing relevance of genetics for their field, they have voiced the need for additional training in genetics; yet, this need is still largely unmet [73]. At the same time, a deep understanding of the spoken and written language phenotypes of individuals with genetic variants is relevant for geneticists/genomicists because the spoken language signal provides sensitive and informative insights into cognitive, motor, and neurological substrates [35, 45, 74–76]. Supporting File SF1 Glossary contains a list of selected technical terms used in this report toward the goal of including a broader and more interdisciplinary readership. The starred and underlined terms in the manuscript are listed alphabetically in the glossary, along with a brief definition.

## 2. Materials and Methods

### 2.1. Participants

Patients 1, 2, and 3, all male, White, and ages 9, 6, and 19 years, respectively, at the time of data collection, and their biological parents participated. In all three cases, family interviews revealed that the three participants were the only individuals in their nuclear and extended families with CAS. Partial findings regarding Patient 1 individually have been previously published in an openly accessible dissertation [77]. Partial findings regarding Patients 2 and 3 individually have also been published in an openly accessible dissertation [78]. This work was conducted with Institutional Review Board oversight at Arizona State University (#STUDY00010292) and the University of Washington (#38237). Parents gave written permission for their minor children to participate, and adults gave written consent to participate. Written parental permission and adult consent included allowing the investigators to use the data for purposes of publication and professional education.

### 2.2. Exome Sequencing and Variant Analysis

Patient 1 and his parents provided saliva samples using OraGene® kits (DNA Genotek, Ottawa, Canada), whereas Patients 2 and 3 and their parents provided peripheral blood samples. DNA was extracted using standard procedures. Exome sequencing was performed at the University of Washington Center for Rare Disease Research (UW‐CRDR). As per the UW‐CRDR standard procedures, the UW‐CRDR processed all receipt, tracking, and quality control/assurance of DNA samples centrally. Initial quality control entailed DNA quantification, sex typing, and molecular “fingerprinting” using a 63‐SNP OpenArray assay derived from a custom exome SNP set. Samples were failed if: (1) the total amount, concentration, or volume was too low; (2) the fingerprint assay produces poor genotype data or integrity of DNA; or (3) sex typing is inconsistent with the sample manifest. Library construction and exome capture have been automated (Perkin–Elmer Janus II) in 96‐well plate format. About 500 ng of genomic DNA is subjected to a series of shotgun library construction steps, including fragmentation through acoustic sonication (Covaris), end‐polishing and A‐tailing, ligation of sequencing adapters, and PCR amplification with dual 10 bp barcodes for multiplexing. All samples for this study passed quality control. Libraries undergo exome capture using the Twist exome (36.5 MB target) (Twist Bioscience). Before sequencing, the library concentration is determined by fluorometric assay and molecular weight distributions verified on the Agilent Bioanalyzer (consistently 180 ± 15 bp). Variant detection and ∗genotyping were carried out with the HaplotypeCaller tool from GATK (4.2.0.0). All exome variants were aligned to the GRCh38/hg38 reference genome.

Annotations were based on SeattleSeq Annotation Server (https://gvs.gs.washington.edu/SeattleSeqAnnotation/). Variants were filtered in seqr [79]. Annotations included allele frequencies in gnomAD (https://gnomad.broadinstitute.org/), Combined Annotation Dependent Depletion (CADD) [80], which is a combined measure of predicted deleteriousness, and ∗“probability of loss‐of‐function intolerance” (plI) with a range of [0, 1] where scores ≥ 0.9 indicate extreme genic intolerance; one nonfunctional gene copy alone is enough to produce an effect [81]. We filtered out low genotyping quality, CADD scores ≤ 10, impact severity scores labeled with LOW, and gnomAD ∗minor allele frequencies ≥ 0.05 [82]. Exomes were searched for *de novo*, ∗autosomal recessive, and ∗x‐linked recessive modes of inheritance. Variants were compared to reported cases in ClinVar (https://www.ncbi.nlm.nih.gov/clinvar/) and DECIPHER [83] and evaluated for potential pathogenicity following established procedures [84, 85].

CNVs were investigated using two complementary methods, Manta Structural Variant Caller [86] and Copy Number Inference from Exome Reads (CoNIFER) [87]. Findings were compared for consensus.

The Integrative Genomics Viewer (IGV) [88] Version 2.16.0 was used to validate all variants. To be validated as *de novo*, a variant had to be absent in both parents and present in approximately half of the offspring’s reads, consistent with ∗heterozygosity in the offspring. The presence or absence of CNVs was also confirmed with IGV.

### 2.3. Genes of Interest for Disorders of Spoken and Written Language

To identify overlaps between genetic pathways and phenotypic expressions in CAS and other relevant conditions, the list of genes of interest from a previous CAS variant study [13] was consulted. This list consisted of 2145 genes assembled from previous CAS studies and also genes implicated in disorders observed to be concomitant with CAS. Kaspi et al. collated this list from the following sources: previously published CAS cohort studies [11, 18]; previously identified single genes in individuals with CAS, high‐confidence genes associated with intellectual disability, epilepsy, and cleft palate; genes included in the SFARI database; brain‐expressed genes associated with primate‐human accelerated evolution that overlap ∗human accelerated regions (HARs); and genes overlapping with HARs that are only expressed in human brains, not in the brains of other primates [89, 90]. For the purposes of this study, two novel genes identified in Kaspi et al.’s [13] study were added to this list, arriving at a set of 2147 genes of interest (Supporting Table ST1). The table from Kaspi et al.’s [13] study is licensed under the Creative Commons Attribution 4.0 International License (https://creativecommons.org/licenses/by/4.0/) that provides unrestricted use, distribution, modification, and reproduction in any medium.

### 2.4. BrainSpan Gene Expression Database

The BrainSpan database in the Allen Brain Atlas at https://www.brainspan.org [91] contains RNA‐Seq data in units of RPKM from 42 brain samples ranging in age from 8 postconception weeks through age 40 years. Of these, 25 samples were derived from ages 8 postconception weeks to postnatal age 10 months, a developmental window considered crucially important for disorders of speech, language, and reading [11, 13, 18]. To evaluate the hypothesis that CAS results from gene disruptions that influence cerebellar development, genes with greatest evidence of pathogenicity in this study were checked for the earliest (prenatal and up to age 10 months postnatal) and later (1–40 years) expression profiles in the cerebellum and all other brain structures combined.

### 2.5. PPI and Functional Module Networks

PPIs and other interactions were retrieved from STRING (https://string-db.org), HPRD (https://ngdc.cncb.ac.cn/databasecommons/database/id/1383), BioGRID (https://thebiogrid.org/), and WikiPI (https://severus.dbmi.pitt.edu/wiki-pi/) databases. GO term enrichment was carried out with Panther (https://geneontology.org/docs/go-enrichment-analysis/). Transcription factor target genes were identified with ChEA3 (https://maayanlab.cloud/chea3/). Protein structures were predicted with αFold3 (https://alphafoldserver.com/) and visualized with PyMOL (https://www.pymol.org/).

## 3. Results

### 3.1. Patient 1

#### 3.1.1. Clinical Background

Patient 1 was a male age 9; 3 (years; months) at the time of data collection. He was born at 39 weeks of gestation via planned cesarean section with a birth weight of 3581 g. The child’s mother reportedly used levothyroxine during pregnancy secondary to hypothyroidism. As per the parent report, infancy was negative for feeding problems, sleeping problems, ear infections, abnormal head size, or other medical complications. No delays were reported in gross or fine motor development, although toilet training was delayed (not fully completed until 5 years of age). Babbling in infancy was limited. First words were delayed, occurring at 24 months of age, and first sentences emerged at age 5 years.

Early intervention for speech and language development was initiated at age 27 months. At this time, Patient 1’s communication abilities were characterized by delayed receptive and expressive language skills, and single words were beginning to emerge. Expressive vocabulary was estimated to be approximately 10 words at this age, whereas typical children produce ∼300 words. Patient 1 was diagnosed with CAS at age 4; 2 based on a limited speech sound inventory, inconsistent vowel production, low intelligibility, and inconsistent speech sound production.

Assessment at age 9; 3 included standardized measures of speech articulation using the Goldman–Fristoe Test of Articulation 2 (GFTA‐2) [92], untimed reading of words and nonwords using the Woodcock–Johnson Tests of Achievement (WJTA) [93], spelling using the Wechsler Individual Achievement Test–Third Edition (WIAT‐III) [94], nonword repetition using the Comprehensive Test of Phonological Processing (CTOPP) [95], expressive and receptive language using the Clinical Evaluation of Language Fundamentals–Fifth Edition (CELF‐5) [96], rapid repetition of mono‐, di‐, and trisyllables [97], a composite measure of fine and gross motor function that taxes cerebellar functions using the Bruininks–Oseretsky Test of Motor Proficiency–2, Brief Form (BOT‐2 BF) [98], and verbal and nonverbal cognition using the Kaufman Brief Intelligence Test‐2 (KBIT‐2) [99].

Results of behavioral testing (Table 1) were consistent with low expressive and receptive language skills as well as low literacy abilities in the presence of low average verbal and nonverbal cognition. Speech production was severely disordered. Speech traits consistent with CAS included vowel errors [(pɛg) for (pɪg)], voicing errors [(su) for (zu)], inconsistent production of words [(saɪdʌ) and (paɪdʌ) for “spider”], vowel additions [(wɪŋʌ) for (rɪŋ)], dysprosody (flat intonation), oral groping, difficulty with multisyllabic words, and incorrectly placed lexical stress (“lion” with stress on the second syllable and “guitar” with stress on the first syllable). Patient 1 also replaced/r/with [w] or vowels, which is a pattern commonly observed in typically developing children at younger ages. Patient 1 averaged DDK *z-*scores of 0.23 for monosyllables and −3.15 for disyllables. Note that a lower score in the disyllables, compared to the monosyllables, is particularly indicative of CAS. Patient 1 obtained a composite fine/gross motor result measured with the BOT‐2 BF that was far below normal limits, indicative of poor cerebellar functions.

**TABLE 1:** Clinical profiles of the three patients.

Genetic variants	Patient 1	Patient 2	Patient 3
Age at most recent assessment	9 years 3 months	6 years 2 months	19 years 2 months
Sex	M	M	M
Birth history	Full term	34 weeks	35 weeks (twin birth)
Hearing history	No concerns	PE tubes before age 18 months	No concerns
Babble history	Sparse	Delayed until age 3 years	Sparse
Age at first words	24 months	48 months	30 months
Cognition: verbal^∗^	−1.20	1.27	1.27
Cognition: nonverbal^∗^	−1.07	0.80	N/A
Speech sound production^∗^	−4.00	−4.00	−2.00^∗∗^
Expressive language^∗^	−3.00	N/A	1.33
Receptive language^∗^	−3.00	Reported to be typical	N/A
Sight word reading^∗^	−2.47	N/A	−0.73
Nonword reading^∗^	−2.60	N/A	−0.73
Spelling^∗^	N/A	N/A	1.33
Monosyllable repetition^∗^	0.23	Too low to score	−1.43
Disyllable repetition^∗^	−3.15	Too low to score	−1.32
Dx of dysarthria	N/A	Yes	No
Gross and fine motor function^∗^	−2.00	Clinically delayed	Clinically delayed
Autism spectrum disorder	Soft signs	Soft signs	Dx as adult

^∗^Numbers represent *Z*‐scores.

^∗∗^Per clinical report at age 6.

#### 3.1.2. Genetic Findings

**FIGURE 1:**
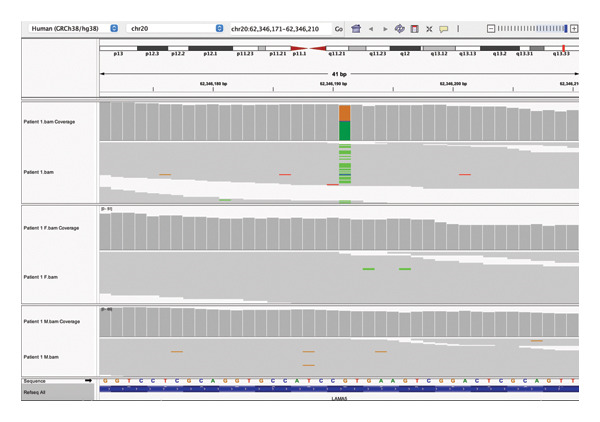
*LAMA5 de novo* variant visualized in IVG, with coverage and .bam tiers for proband, father, and mother shown from the top to the bottom tiers.

No CNVs of plausible influence on the phenotype were found. Only one *de novo* variant remained after filtering, a transition missense variant (c.1307C>T, p.Thr436Met) located in exon 10/80 of the *LAMA5* (laminin alpha 5) gene, OMIM ∗601033, on 20q13.33 at position chr20: 62,346,191[hg38]. It is rare in the gnomAD exomes (1.2^−5^). The CADD score is 21.9, and the variant is predicted to be damaging by multiple algorithms. This variant fits the PS2 (*de novo* status confirmed with both parents tested) and PM3 (absent from controls) categories in the ACMG standards, resulting in a “likely pathogenic” classification. For further information about this and all other variants in this study, see Table 2. Figure 1 shows the IGV visual check for *de novo* inheritance as an example of all IGV checks performed to validate variants of interest.

**TABLE 2:** Exome variants and annotations for each of the three patients.

P	Inh.	Chr.	Pos.	Gene	OMIM	Molecular pathway and function	pLi	Effect	gnomAD exomes freq.	CADD	sift	fathmm	rsid	Transcript DNA variant	Transcript prot. change
1	*DN*	20	62,346,191	**LAMA5**	601033	ECM glycoprotein; integrin signaling; cell adhesion; no direct gene regulation role	0	Missense	1.2*E* − 05	21.9	Damaging	Damaging		ENST00000252999.7:c.1307C>T	ENSP00000252999.3:p.Thr436Met
	AR	6	111,372,921	REV3L	602776	Translesion DNA synthesis, DNA repair and genome stability; indirect transcription regulation role	1	Missense	1.4*E* − 02	18.5	Tolerated	Damaging	rs3218599	ENST00000358835.7:c.5434G>C	ENSP00000351697.3:p.Asp1812His
		16	2,964,989	KREMEN2	609899	Wnt pathway receptor; modulates beta‐catenin signaling; indirect transcriptional role	0	Missense	1.7*E* − 02	17.3	Tolerated		rs62032326	ENST00000303746.9:c.225C>A	ENSP00000304422.5:p.Ser75Arg
		16	27,749,614	KATNIP (KIAA0556)	616650	Ciliogenesis and microtubule regulation; no known gene regulation function	0	Missense	1.4*E* − 02	22.6	Tolerated		rs16976970	ENST00000261588.8:c.2654G>A	ENSP00000261588.4:p.Arg885Gln
		19	8,605,262	ADAMTS10^∗^	608990	ECM metalloprotease; microfibril remodeling; no direct gene regulation function	1	Missense	3.1*E* − 02	23.2	Tolerated	Damaging	rs62621197	ENST00000270328.8:c.185G>A	ENSP00000270328.4:p.Arg62Gln
	XLR	X	14,911,253	MOSPD2	301086	ER membrane contact sites and immune cell migration; no direct gene regulation role		Missense	2.6*E* − 02	23.5	Damaging	Damaging	rs35164803	ENST00000380492.7:c.719G>A	ENSP00000369860.3:p.Ser240Asn
		X	101,841,958	NXF5	300319	Nuclear RNA export factor; mRNA transport; indirect gene expression regulation role		Missense	1.9*E* − 02	23.0	Damaging	Damaging	rs55756985	ENST00000473265.2:c.67A>G	ENSP00000426978.1:p.Lys23Glu
		X	120,605,465	MCTS1	300587	Translation re‐initiation factor; regulates protein synthesis; indirect gene regulation role		Missense	7.4*E* − 04	26.8	Damaging	Damaging	rs143806871	ENST00000371315.3:c.73G>T	ENSP00000360365.3:p.Gly25Cys
		X	131,285,348	IGSF1	300137	Transmembrane protein; pituitary hormone regulation; possible indirect transcriptional effects		Missense	4.8*E* − 04	22.7	Tolerated		rs201255931	ENST00000370903.7:c.498G>C	ENSP00000359940.3:p.Glu166Asp
		X	141,897,457	MAGEC3	300469	Cancer/testis antigen; apoptosis signaling; no direct gene regulation role		Missense	4.7*E* − 03	10.6	Tolerated		rs143730187	ENST00000298296.1:c.1699G>T	ENSP00000298296.1:p.Val567Phe

2	*DN*	12	109,285,446	**FOXN4**	609429	Transcription factor (Forkhead family); regulates neurodevelopmental genes	1	Synonymous	0	17.9				ENST00000299162.9:c.759G>A	ENSP00000299162.5:p.Lys253=
	AR	1	200,873,513	GPR25	602174	GPCR for chemokine ligand CXCL17; immune cell trafficking; indirect regulation of gene expression via second messenger signaling	0.005	Missense	1.6*E* − 02	21.0	Tolerated		rs144298915	ENST00000304244.4:c.476C>T	ENSP00000301917.2:p.Ser159Leu
		5	141,641,727	FCHSD1	617555	Actin cytoskeleton and endocytosis regulator; no direct gene regulation role	0	Missense	2.0*E* − 02	23.2	Tolerated	Damaging	rs116772138	ENST00000435817.6:c.1982A>C	ENSP00000399259.2:p.Asp661Ala
		10	7,171,965	SFMBT2	615392	Chromatin remodeler, polycomb‐group chromatin protein, transcriptional repression	1	Missense	2.3*E* − 03	24.4	Tolerated	Damaging	rs201963815	ENST00000397167.5:c.2345G>A	ENSP00000380353.1:p.Arg782His
		15	98,971,106	PGPEP1L		Peptide processing enzyme; no gene regulatory role	0	Missense	1.1*E* − 02	23.7	Tolerated	Damaging	rs116915938	ENST00000378919.6:c.74T>C	ENSP00000368199.6:p.Leu25Pro
		19	34,377,871	GPI	172400	Glycolytic enzyme and neurotrophic factor; indirect metabolic influence on gene expression	0	Missense	1.6*E* − 02	24.7		Damaging	rs8191371	ENST00000588991.7:c.656T>C	ENSP00000465858.3:p.Ile219Thr
	XLR	X	3,076,561	ARSF	300003	Lysosomal sulfatase; glycosaminoglycan degradation; no gene regulation role		Missense	4.5*E* − 04	23.0	Damaging	Damaging	rs111289343	ENST00000381127.5:c.175G>C	ENSP00000370519.1:p.Asp59His
		X	21,992,652	SMS^∗^	300105	Polyamine biosynthesis; chromatin structure modulation via polyamines; indirect gene regulatory effect		Missense	4.9*E* − 05	15.2	Tolerated	Damaging	rs199670083	ENST00000404933.6:c.1001G>A	ENSP00000385746.2:p.Arg334His
		X	100,850,206	NOX1	300225	ROS‐generating enzyme; modulates NF‐kB signaling; indirect transcriptional regulation		Missense	1.9*E* − 02	22.9	Damaging	Damaging	rs34688635	ENST00000372966.7:c.1078G>A	ENSP00000362057.3:p.Asp360Asn

3	*DN*	8	12,138,512	**USP17L2**	10186	Deubiquitinase; stabilizes signaling proteins; potential indirect transcriptional effects	0	Missense	1.1*E* − 03	10.7	Tolerated		rs200186296	ENST00000333796.3:c.249G>C	ENSP00000333329.3:p.Gln83His
		12	40,278,097	**LRRK2**	609007	Kinase involved in vesicle trafficking and inflammation; regulates NF‐kB and interferon	0	Missense	0	19.7	Damaging	Damaging		ENST00000298910.11:c.2077A>C	ENSP00000298910.7:p.Asn693His
	AR	7	107,929,517	**LAMB1** ^∗^	150240	ECM glycoprotein; brain basement membrane formation; no gene regulation role	0	Missense	1.9*E* − 02	27.3	Damaging	Damaging	rs35915664	ENST00000222399.10:c.4640T>C	ENSP00000222399.6:p.Ile1547Thr
		17	10,309,647	MYH13	603487	Myosin motor protein (extraocular/laryngeal muscles); no gene regulatory role	0	Missense	1.4*E* − 02	24.4		Damaging	rs35069886	ENST00000418404.8:c.4840G>C	ENSP00000404570.3:p.Asp1614His
		22	26,623,300	CRYBA4	123631	Structural lens protein; no gene regulatory role	0	Missense	1.7*E* − 02	23.6	Damaging		rs35520672	ENST00000354760.3:c.106G>A	ENSP00000346805.3:p.Val36Met
	XLR	X	18,954,379	PHKA2	300798	Phosphorylase kinase regulatory subunit; glycogen metabolism; no gene regulation role		Missense	2.0*E* − 02	18.8	Tolerated	Damaging	rs17313469	ENST00000379942.4:c.112G>C	ENSP00000369274.4:p.Glu38Gln
		X	70,258,582	P2RY4	300038	Receptor for UTP and UDP coupled to G proteins; indirect regulation via second messengers		Stop gained	1.3*E* − 02	36.0			rs41310667	ENST00000374519.3:c.1043G>A	ENSP00000363643.2:p.Trp348Ter
		X	136,414,167	ADGRG4	301085	Adhesion GPCR; gut neuroendocrine signaling; no direct gene regulatory role		Missense	3.3*E* − 03	16.0	Damaging		rs140250794	ENST00000394143.5:c.9045C>A	ENSP00000377699.1:p.Ser3015Arg
		X	152,945,346	ZNF185	300381	Actin‐associated LIM domain protein; cytoskeletal scaffolding; no gene regulatory role		Missense	3.3*E* − 04	13.4	Tolerated		rs377079307	ENST00000535861.5:c.1384G>A	ENSP00000440847.1:p.Gly462Ser

*Note:* Bold font: gene of highest confidence.

Abbreviations: AR, autosomal recessive; *DN*, *de novo*; P, patient; XLR, X‐linked recessive.

^∗^Included in the adapted list of genes of interest compiled by Kaspi et al. [13].

**FIGURE 2:**
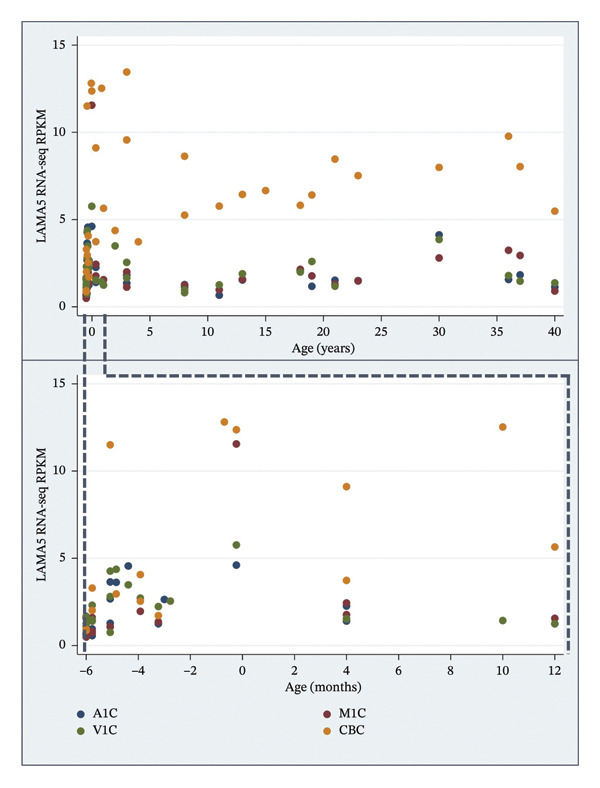
*LAMA5* RNA‐seq RPKM in four selected brain regions for ages postconceptual 8 weeks to 40 years and separately for prenatal and postnatal age up to 12 months. A1C, primary auditory cortex; CBC, cerebellar cortex; M1C, primary motor cortex; V1C, primary visual cortex.

The BrainSpan database shows *LAMA5* expression levels averaged across all available brain structures of 2.30 RPKM for the earliest (prenatal and up to 10 months postnatal) and 2.15 RPKM for later (ages 1 through 40 years) ages. Expression levels in the cerebellum are 5.74 and 7.17 for these two age ranges, respectively, with both cerebellar expression rates higher than in the other brain regions averaged (Table 3). Figure 2 shows higher *LAMA5* expression rates in the cerebellum, compared to primary motor, auditory, and visual cortices across all available ages in BrainSpan, consistent with H4 (cerebellar locus of impairment).

**TABLE 3:** Gene expression rates in early (prenatal up to 12 months postnatal) and older samples, separately cerebellum and average of all other brain structures based on BrainSpan data.

Patient	Inheritance	Gene	Average RPKM < 12 m	Average RPKM ≥ 12 m	Cerebellum RPKM < 12 m	Cerebellum RPKM ≥ 12 m
1	de novo	**LAMA5**	2.3	2.15	5.74+	7.17+
	Aut. rec.	REV3L	5.93	2.2	7.39+	5.94+
		KREMEN2	0.29	0.88	0.17	0.14
		KATNIP	2.22	2.14	2.64+	1.75−
		ADAMTS10	7.47	2.64	5.84−	0.77−
	X‐linked rec.	MOSPD2	1.81	3.61	2.50+	2.63+
		NXF5	0.01	0.02	0	0.02
		MCTS1	9.79	7.93	6.80−	4.12−
		IGSF1	1.72	4.49	2.17+	1.27−
		MAGEC3	0.06	0.14	0.09	0.03

2	de novo	**FOXN4**	0.86	0.03	0.67	0.04
	Aut. rec.	GPR25	0.10	0.03	0.14	0
		FCHSD1	1.85	3.66	2.87+	4.65+
		SFMBT2	1.67	1.78	4.93+	2.36+
		PGPEP1L	0.05	0.13	0.05	0.02
		GPI	35.96	55.1	38.31+	70.11+
	X‐linked rec.	ARSF	0.87	1.6	0.32	0.19
		SMS	19.77	10.17	11.41−	11.81+
		NOX1	0.17	0.18	0.21	0.29

3	de novo	**USP17L2**	0.01	0.02	0.01	0.01
		**LRRK2**	0.49	1.73	0.57	0.62
	Aut. rec.	**LAMB1**	5.61	3.85	11.82+	7.60+
		MYH13	0.01	0.02	0.02	0
		CRYBA4	0.05	0.1	0.1	0
	X‐linked rec.	PHKA2	2.64	1.45	2.81+	2.26+
		P2RY4	0.01	0.01	0.02	0.01
		ADGRG4	0	0	0.01	0
		ZNF185	1.81	1.19	2.60+	1.06−

*Note:* Bold font: gene of highest confidence. +Expression levels higher in the cerebellum than in all other brain regions averaged (genes with RPKM > 1 in the earliest development). −Expression levels lower in the cerebellum than in all other brain regions averaged (genes with RPKM > 1 in the earliest development).

The *LAMA5* variant was selected as a gene of high priority based on the following additional supporting evidence: According to the NCBI Entrez gene summary (https://www.ncbi.nlm.nih.gov/), *LAMA5* encodes one of the vertebrate laminin alpha chains. Laminins are implicated in several cellular processes including cell adhesion, differentiation, migration, signaling, neurite outgrowth, and metastasis. A *de novo LAMA5* variant (c.10828+1G>A; *p*. unknown) was found in a Korean child with substantially delayed speech, language, and motor development and limited social skills [100]. A patient with a ∗homozygous *LAMA5* variant (c.8046C>T, p.Arg2659Trp) had a severe neuromuscular transmission failure and mildly reduced cerebellar volume [101]. Whereas the list of genes of interest collated by Kaspi et al. [13] does not include *LAMA5*, it contains two other laminin genes (*LAMB1* and *LAMB2*) that are all in close functional relationship with *LAMA5*.

Further, Patient 1 carried four variants inherited via autosomal recessive inheritance (Table 2). Of the four implicated genes, three had average RPKM expression rates > 1 in the earliest developing brain. The variant in a gene with the highest average expression rate in the early developing cerebellum was rs3218599 in the *REV3L* gene (REV3‐like, DNA‐directed polymerase zeta catalytic subunit, OMIM ∗602776), earliest RPKM 7.39 and 5.93, in the cerebellum and whole brain average, respectively, consistent with H4 (cerebellar locus of impairment).

Five X‐linked recessive variants for which the mother was ∗heterozygous and the proband ∗hemizygous met filtering criteria. Of these, three were situated in genes with RPKM scores of > 1. For two of these, *MOSPD2* and *IGSF1*, expression rates in the early developing cerebellum were higher than in all regions combined, albeit by a small amount.

To summarize, the main variant of interest for Patient 1 was the rare *de novo* missense variant in *LAMA5*, here described as likely pathogenic. To what extent other variants contributed to the phenotype was investigated with functional tools, described below.

### 3.2. Patient 2

#### 3.2.1. Clinical Background

Patient 2 was a male, age 6; 2 at the time of data collection. His older brother was typically developing. As per the medical report, Patient 2 had a history of developmental delay, hypotonia, macrocephaly, hydrocephaly, severe upper respiratory infections, allergy to sulfa drugs, and eczema. Prenatal and birth history provided by parents indicate that the mother had complete placenta previa and low amniotic fluid. Patient 2 was born prematurely at 34 weeks of gestation and was hospitalized for 7 days. He had difficulty nursing and had frequent respiratory and ear infections, necessitating the placement of pressure equalization tubes twice before the age of 18 months. Patient 2 had a history of visuospatial processing deficits. Delays in fine and gross motor development were addressed with occupational and physical therapy between ages 2 and 5 years. As a preschooler, he was diagnosed with ASD, but this diagnosis was later modified to state that he showed soft signs of ASD.

Developmental milestones for speech and language were delayed, with babbling emerging around age 3, first words around age 3; 6 and sentences at age 4; 6. At age 6 years, he struggled significantly with learning to read and spell, in particular by mis‐sequencing letters in words. He was diagnosed with both CAS and dysarthria. He had received speech‐language therapy since age 2 years. He previously participated in genetic testing to evaluate the possibility of fragile X and ASD. Genetic testing for *FMR1*, *FMR2*, and *PTEN* variants resulted in no significant findings.

Assessment measures at age 6; 2 included speech articulation testing using the GFTA‐2 [92] and nonverbal and verbal IQ testing using the Reynolds Intelligence Assessment Scales (RIAS) [102]. Results (Table 1) showed typical verbal and nonverbal IQ but severely delayed speech. Speech errors consistent with CAS were vowel errors, mis‐sequenced sounds, and inconsistently produced words. For instance, the word “yellow” was produced as [lowəl] and [loʔo], illustrating both inconsistency and mis‐sequencing of sounds.

#### 3.2.2. Genetic Findings

As with Patient 1, no plausible CNVs were found. A *de novo* variant was a synonymous change in *FOXN4* (Forkhead box 4N), OMIM ∗609429, at position chr12:109,285,446 [hg38] (c.759C>T, p.Lys253=), no rsID, CADD = 17.9. This variant is absent in the gnomAD exome database and not listed in ClinVar. The variant fulfills PM2 and PS2 criteria for likely pathogenic status. *FOXN4* is not expressed highly in the brain at any age. It is, however, a DNA‐binding transcription factor essential for neural development and is situated in a highly conserved region with a PhyloP score of 2.13. For these reasons, it was considered to be of high interest.

Further, Patient 2 carried five autosomal recessive variants. The variant in a gene with highest expression rates in the cerebellum was rs8191371, a missense variant in *GPI* (glucose‐6‐phosphate isomerase), OMIM ∗172400, and the CADD score is 24.7. Early cerebellar and whole brain expression rates are 38.31 and 35.96, respectively, consistent with H4 (cerebellar locus of impairment).

Three X‐linked recessive variants were found. Of these, only one is situated in a gene expressed in the early developing brain, *SMS* (spermine synthase), OMIM ∗300105, a gene associated with intellectual developmental disorder, X‐linked, syndromic, Snyder–Robinson type. Earliest cerebellar and whole brain RPKM rates are 11.48 and 19.77, respectively. Of note, this gene is listed among the genes of interest in Kaspi et al. [13] as implicated in clefting, genetic epilepsy, and intellectual disability.

To summarize, the main gene of interest is *FOXN4*. Additional genes with variants that met filtering criteria were included in functional analyses.

### 3.3. Patient 3

#### 3.3.1. Clinical Background

Patient 3 was a male age 19; 2 at the time of data collection. As per the parent report, he and his brother are monozygotic twins and have highly similar clinical histories, but only he participated in this study. Prenatal and birth history indicate that the twins were delivered 5 weeks prematurely via cesarean section. Patient 3’s developmental milestones were reported to be within normal limits except for speech and language skills. Babbling was minimal to absent, and first words were not produced until around 3 years of age, at which time he began receiving intermittent speech‐language therapy. Testing at age 6; 6 revealed a variety of articulation and phonological errors along with instances of articulatory imprecision and voicing errors. Additionally, vowel distortions during a DDK task and difficulty with the production of multisyllabic words were noted. Prosody was reported to be unusual, characterized by monotone speech and prolongations occurring both within and between word and syllable junctures, significantly impacting his intelligibility. Oral motor skills were delayed and characterized by exaggerated movements. These data indicate difficulty with motor planning and traits associated with CAS. Though initially presenting with an expressive language delay at age 2; 6, his receptive and expressive language skills were reported to be within normal limits at 6; 6. Patient 3 received school‐based speech therapy from 1^st^ through 6^th^ grade. He was diagnosed with high‐functioning autism as a college student.

At age 19; 2, behavioral testing (Table 1) included verbal and nonverbal IQ assessment using the RIAS [102], expressive language testing using the Recalling Sentences subtest from the Clinical Evaluation of Language Fundamentals–4 (CELF‐4) [103], sight word reading and nonword decoding testing using the Word Identification and Word Attack subtests, respectively, from the Reading Mastery Tests–Revised (WRMT‐R; [93]), and spelling skills using the Wechsler Individual Achievement Test 2 [104]. Performance on all of these metrics was within normal limits. Monosyllabic and disyllabic syllable repetition speeds were < −1 SD, however, and his conversational speech was characterized by slow‐speed, robotic rhythm, and monotone intonation, traits that were likely residual effects of CAS.

#### 3.3.2. Genetic Findings

**FIGURE 3:**
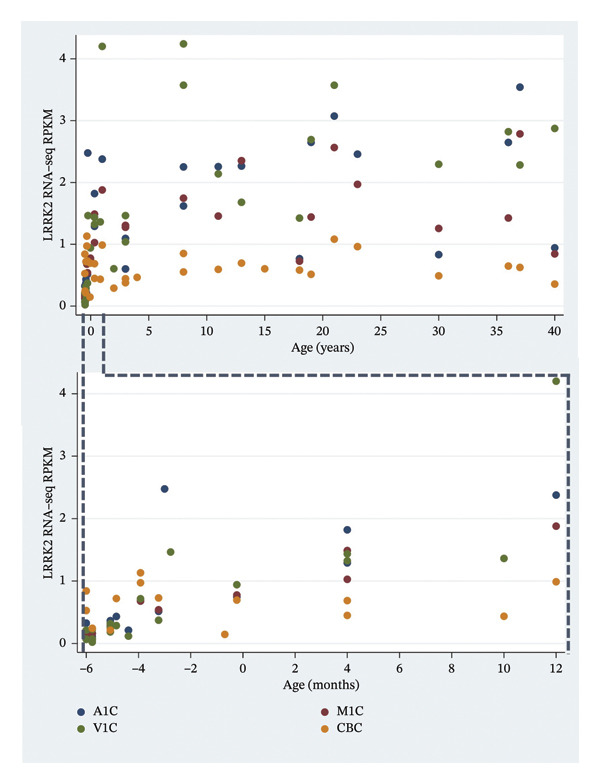
*LRRK2* RNA‐seq RPKM in four selected brain regions for ages postconceptual 8 weeks to 40 years and separately for prenatal and postnatal age up to 12 months. A1C, primary auditory cortex; CBC, cerebellar cortex; M1C, primary motor cortex; V1C, primary visual cortex.

As with the other two patients, no plausible CNVs were found. Exome analysis revealed two *de novo* missense variants. One is rs200186296, situated in *USP17L2* (ubiquitin‐specific peptidase 17‐like family member 2), OMIM ∗610186, also referred to as *DUB3*, at position chr8: 12138512 [hg38]. The c.249G>C substitution leads to a p.Gln83His amino acid substitution, with a CADD score of 10.7. This variant is rare in the gnomAD population (allele frequency = 0.001). *USP17L2* regulates several cellular processes including signal transduction and has potential indirect transcriptional effects. It has been implicated in the molecular pathway of glioblastoma multiforme [105]. According to the BrainSpan database, it is not highly expressed in any brain region at any age. The *de novo* status of this variant (PS2) and its low frequency in the reference population (PM3) qualify for a “likely pathogenic” classification, and although not annotated as damaging, it was included as a variant of high interest because of the gene’s potential indirect transcriptional effects. The second *de novo* variant is situated in *LRRK2* (leucine‐rich repeat kinase 2), OMIM ∗609007, at position chr12: 40278097 [hg38], no rsID. This c.2077A>C substitution results in a p.Asn693His amino acid substitution, with a CADD score of 19.51. It is absent from the reference population and annotated as damaging. *LRRK2* encodes a large multidomain protein kinase. It is associated with Parkinson’s disease 8, autosomal dominant, and hereditary late‐onset Parkinson’s disease. According to the BrainSpan database, this gene has relatively low expression rates in the brain across the lifespan. The *de novo* status of this variant (PS2) and its absence from the reference population (PM3) qualify for a “likely pathogenic” classification. The role of *LRRK2* in neurogenesis [106] was an additional consideration for inclusion in the list of high‐priority genes. Figure 3 shows *LRRK2* expression rates in four selected brain regions across the lifespan.

**FIGURE 4:**
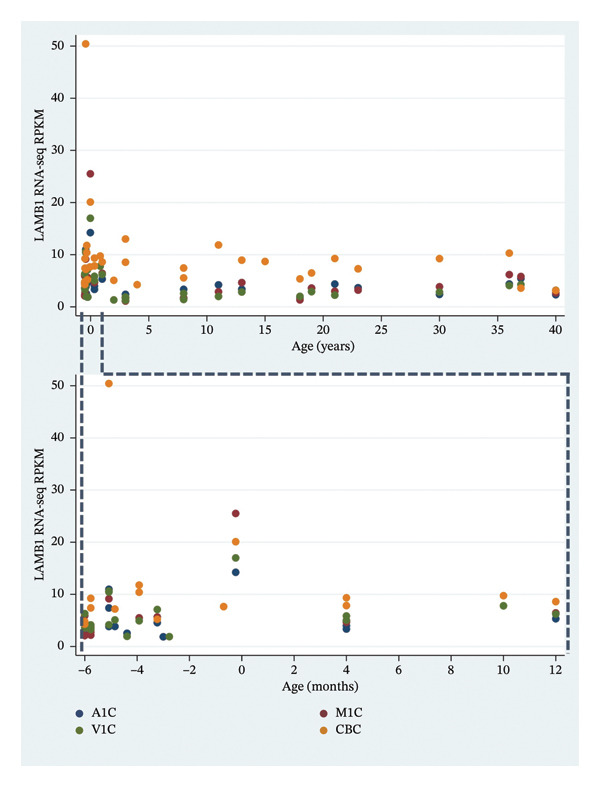
*LAMB1* RNA‐seq RPKM in four selected brain regions for ages postconceptual 8 weeks to 40 years and separately for prenatal and postnatal age up to 12 months. A1C, primary auditory cortex; CBC, cerebellar cortex; M1C, primary motor cortex; V1C, primary visual cortex.

Patient 3 also carried five autosomal recessive variants. A transition missense variant is situated in *LAMB1* (laminin beta subunit 1), OMIM ∗150240. The CADD score is 28.50. This variant is annotated as damaging. *LAMB1* is expressed more highly in the earliest cerebellum than the average of the whole brain, with RPKM of 11.82 and 5.61, respectively, consistent with H4. Figure 4 shows the RPKM values throughout the lifespan for the cerebellum and three other brain structures.

This variant was considered to be of high interest for the following additional reasons: Like *LAMA5*, *LAMB1* is a member of the laminin genes that influence cell adhesion, migration, and neurite outgrowth. Associated diseases are ASD, lissencephaly 5, and Pierson syndrome. *LAMB1* physically interacts with *LAMA5*, in the laminin‐511 complex, for which a structure has been experimentally determined, in the presence [107] and absence [108] of its integrin receptor through which it signals [109]. It is included in both the SFARI list of autism genes and Kaspi et al.’s [13] list of genes of interest.

Further, Patient 3 carried four X‐linked recessive variants. Of these, one is a missense variant situated in the *ZNF185* gene (zinc finger protein 185 with LIM domain, OMIM ∗300381) (c.1384G>A, p.Gly462Ser). Although the CADD score is high (36), gene expression in the brain is minimal, with the highest RPKM of 2.6 seen in the early developing cerebellum.

To summarize the genetic findings for Patient 3, the role of the *USP17L2 de novo* variant, while fulfilling PS2 and PM2 criteria, is uncertain due to its low expression rates in brain and its physiological functions; however, its potential transcriptional effects may elevate its relevance in the context of neurodevelopmental disorders. The *LRRK2 de novo* variant has a high CADD score and is involved in neurodevelopment. Patient 3 is homozygous for a deleterious *LAMB1* missense variant. Given this gene’s role in neurogenesis, it is possible that this variant contributed to the phenotype. All variants that met filtering criteria were included in the functional investigation, described below.

### 3.4. Aggregate Findings Across Participants

#### 3.4.1. Phenotypes and Genotypes

Table 1 summarizes the clinical phenotypes in the three patients. Note the similarities in the sparse babble history, late onset of words, low scores in disyllable repetition, delays in fine and gross motor development, and signs of ASD, consistent with shared core and associated traits. Only Patient 1 had delayed verbal and nonverbal IQ and struggled with written language. Performance on written language tasks was not available for Patient 2.

Table 2 is a summary of the exome variants found in the three patients. Note that of the 28 genes of interest in this study, three are also included in the list of 2147 genes adapted from Kaspi et al.’s [13] study, namely *ADAMTS10* (implicated in ID), *LAMB1* (implicated in ID and listed in the SFARI database as a strong ASD candidate), and *SMS* (implicated in clefting, genetic epilepsy, and ID).

In the early developmental phase of interest, prenatal to postnatal age 12 months, 11 of 14 genes with early expression rates of > 1 RPKM were expressed more highly in the cerebellum, compared to all other brain regions averaged. This includes two of the five genes of highest confidence, *LAMA5* and *LAMB1*, known members of the same protein complex (see below). Three genes were expressed in the cerebellum at a lower rate than the whole brain average. In the later developmental phase, 1 through 40 years, 9 of 14 genes were expressed more highly in the cerebellum than in the other brain regions averaged, and 5 were expressed at a lower rate. Table 3 summarizes gene expression rates in the early and later brain, separately for the cerebellum and average of other structures.

#### 3.4.2. Functional Interpretation of the Genes of Interest

**FIGURE 5:**
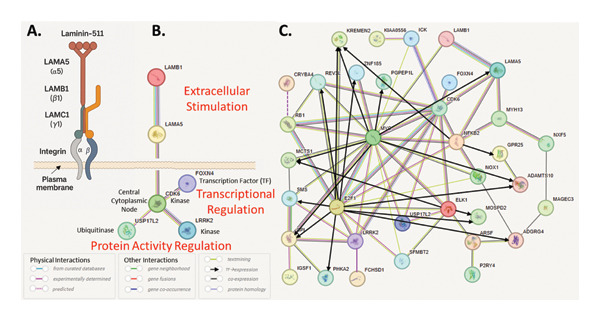
(A) Physical interaction between *LAMA5* and *LAMB1* in the laminin‐511 complex. Laminin‐411 interacts with and activates integrin receptors, which traverse the membrane and transmit the laminin binding signal to the inside of the cell. (B) Network of the five high‐confidence genes, expanded by *CDK6*, a known downstream kinase in laminin‐integrin signaling networks​ [111]. Interactions were retrieved from the STRING database, and the types of interactions represented by their color codes are indicated in the legend below (A, B), described here as follows: Pink indicates direct, experimentally determined, physical PPI, dashed pink indicates HiPPIP [62] predicted PPI, light blue: PPI from curated databases, dark blue: gene co‐occurrence, red: gene fusions, green: gene neighborhoods, yellow: text mining, black: co‐expression, blue: protein homology. (C) Full network of all 28 genes of interest using STRING edges, augmented by transcriptional control of genes (black arrows), retrieved from Chea3 (see Methods). Edge colors are the same as in B. One putative interaction was added (dashed purple line), between *CRYBA4* and *RB1*, predicted by HiPPIP [62].

To understand what is the specific role of the genes identified with variant filtering in child brain development and function that could explain their association with CAS, we first carried out ∗GO term enrichment analysis on the set of five genes of highest confidence as well as the full set of 28 genes of interest but were unable to identify a common functionality, due to the small number of input genes in the first group and the large number of different functions represented overall (data not shown). We therefore analyzed the five high‐priority genes in the context of their known interactions to arrive at a CAS disease network. Guilt by association is a principle frequently used in expanding gene/protein networks based on the assumption that if one protein is involved in a given function, its interaction partners might also be associated with that same function [110]. Figure 5 summarizes interactions among the five genes of highest confidence.

To cast the widest possible net of possible interactions, we relied on the STRING database (https://string-db.org) as a central integrative resource of protein association network evidence. STRING combines several different types of connections (gene expression, physical interaction, etc.) into one network. Of the five high‐confidence genes, only *LAMA5* and *LAMB1* are directly connected in STRING, supported by all types of edges (see edge color codes in Figure 5), including interactions at gene regulatory level as well as physical interactions. This is because there is a large body of evidence that the two proteins are two subunits of trimeric laminin of which there are 16 different types that have been experimentally identified, with the possibility of additional combinations that are theoretically possible but not yet experimentally confirmed [109]. Of these, only laminin‐511 comprises both *LAMA5* and *LAMB1*, which means that the third subunit is most likely *LAMC1* (Figure 5(A)). To identify how these two genes may be connected to the other high‐confidence genes, we systematically tested each of the STRING interactions of all 5 genes to any other gene, and found that it was sufficient to add a single gene, *CDK6*, to connect all five high‐confidence genes to each other (Figure 5(B)). *CDK6* as a central node signaling kinase then connects to transcriptional and protein function regulation through transcription factor activity and post‐translational activity, respectively. CDK6 is a cyclin‐dependent serine/threonine kinase 6 that phosphorylates and inhibits retinoblastoma (RB) protein family members, such as RB1. This in turn regulates cell‐cycle progression and cell differentiation because the phosphorylation of RB1 allows dissociation of the transcription factor E2F from RB/E2F complexes enabling the transcription of E2F target genes. Because Forkhead box protein N4, FOXN4, is also a transcription factor essential for neural tissue development in the central nervous system, we looked for which genes may be under *FOXN4*’s control. This experiment has been done in Xenopus laevis [112]. The data were downloaded from GEO (https://www.ncbi.nlm.nih.gov/geo/query/acc.cgi?acc=GSE89271). Two of the 28 listed genes appear as significantly regulated *FOXN*4 targets in the GSE89271 CRISPR dataset: *PHKA2* and *IGSF1*, with log_2_ fold change of +2.38 (adjusted *p* value 1.5 × 10^−4^) and −5.17 (*p* = 1.6 × 10^−4^), while none of the others were significantly altered (FDR < 0.05) in this dataset. We then checked what other transcription factors may be involved in the regulation of the 28 genes and searched the Che3 database (see Methods). Because there were many, we added to the network only the minimum transcription factors needed to connect all 28 genes (*MYC*, *NFKB*, *ELK1*, and *E2F1*). The black arrows in Figure 5(C) show which genes’ expression levels are controlled by these transcription factors. Finally, only three genes remained unconnected: *FCHSD1*, *CRYBA4*, and *KIAA0566* = *KATNIP*. We identified connections for all three at the protein level through HiPPIP [62]. *FCHSD1* interacts with *LRRK2*. KATNIP is a scaffold protein that binds and stabilizes ICK, enhancing its kinase activity [113]. *CRYBA4* is predicted to interact with *RB1* (controlled by *CDK6*, see above), which in turn connects to several other genes in the network. The result is a densely connected network with 32 nodes and 73 edges (Figure 5(C)), which we predict here is under the control of laminin‐511 (Figures 5(A) and 5(B)). While we did not find a known integrin receptor for laminin‐511 among these proteins, there are several membrane receptors in this list, which could act as the laminin‐511 receptor or co‐receptor (e.g., *GPR25*, a G protein–coupled receptor). Binding of laminin to their cognate receptor(s) is expected to mediate the attachment, migration, and organization of cells into tissues through interaction with other extracellular matrix components during embryonic development, especially the formation of the laminar architecture of the cerebral cortex. This process allows maintaining the integrity of the basement membrane which acts as a physical barrier to migrating neurons essential in cerebral cortical development. As described above, the laminin binding–induced receptor activation will be transmitted to the cytoplasmic side, where it stimulates a signal transduction cascade that likely involves *CDK6*. In addition to transmitting the signal to *FOXN4* (Figure 5(B)) and other (Figure 5(C)) proteins that regulate protein activity through transcription, *CDK6* also connects to protein activity regulation through post‐translational modifications. *CDK6* binds to *USP17L2*, ubiquitin carboxyl‐terminal hydrolase 17, which deubiquitinates proteins involved in cell‐cycle progression, cell proliferation, and apoptosis and thus also indirectly contributes to transcriptional regulation. *CDK6* also binds to *LRRK2*, leucine‐rich repeat serine/threonine‐protein kinase 2, and *MARK2*, microtubule affinity regulating kinase 2, serine/threonine‐protein kinase genes involved in neuronal plasticity, autophagy, and vesicle trafficking and cell polarity and microtubule dynamics regulation during neuronal migration, respectively. LRRK2 as one of the kinases known to phosphorylate tau has been implicated in several neurodegenerative diseases and is currently particularly actively studied to protect neurons in Parkinson’s disease [114].

#### 3.4.3. Molecular Mechanism of Laminin‐511 Mutations in CAS

**FIGURE 6:**
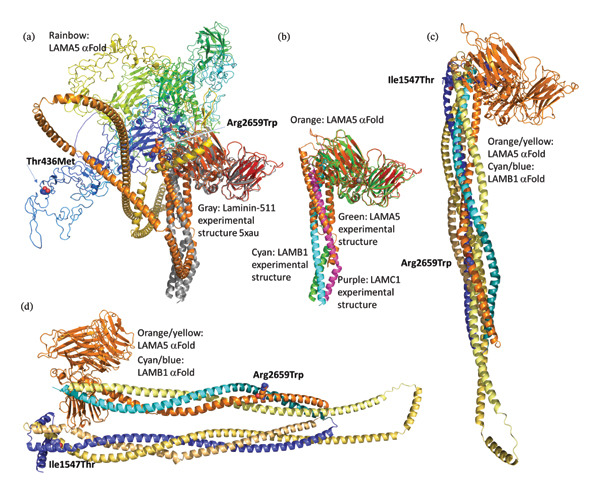
Three‐dimensional structure of laminin‐511 and predicted locations of CAS mutations. (a) Alpha‐fold prediction of LAMA5 superimposed on the experimental structure [108] with pdb id 5xau (gray). To highlight the progression of structure along the sequence, the very N‐terminus is shown in blue, progressing in rainbow toward the C‐terminus. Residues 1–3292 were predicted encompassing the entire N‐terminus before and including the region subjected to cryo‐EM structure determination [108]. (b) Same as (a) but omitting predictions outside of the residues subjected to cryo‐EM. (c) Joint alpha‐fold prediction of LAMA5 helical regions (residues 2170–2705) and LAMB1 (residues 1487–1786). (d) Same as (c), rotated.

Given that we predict a central role for laminin‐511 in the control of the CAS disease network, we studied in greater detail the structure–function relationship of the mutations found in our patients in LAMA5 and LAMB1. A cryo‐EM structure is available in the protein databank under pdb id 5xau for the core of laminin‐511 containing 674 of the 7390 amino acids of LAMA5, plus one helix each from LAMB1 (74‐amino‐acid fragment, at the very C‐terminus of the 1786 long sequence) and LAMC1 (83‐amino‐acid fragment), respectively. Figure 6(a) shows this structure in gray, superimposed with the αFold prediction (see Methods) of the missing N‐∗terminus including the region included in the 5xau structure of the LAMA5 sequence (3292 out of 7390 amino acids), shown in rainbow colors from blue (N‐terminus) to red (C‐terminus). The two mutations of interest in *LAMA5*, Thr436Met (Table 2) and Arg2659Trp mentioned in a case with neuromuscular and cerebellar anomalies [101], lie outside of the experimentally determined region. Their predicted locations are indicated by arrows. For comparison, the 5xau experimentally determined structure is shown in Figure 6(b), omitting the regions of LAMA5 that were not visible by cryo‐EM, and highlighting the three subunits in their respective colors. As we can see, the predicted structure can be divided into two regions, residues 1–2169, which are globular and largely comprised of EGF‐like repeat domains and where Thr436Met is located, and residues 2170–2705, which are entirely helical and carry the Arg2659Trp mutation. The way the helices are placed randomly in space with little contact to the remainder of the structure and that when predicted in isolation it takes on the structures of the β and γ‐subunits (note the prediction in orange where the two subunits are located in 5xau (Figure 6(b)) strongly indicate that while the secondary structure prediction of helix is accurate, the placement in three‐dimensional space is not. This finding supports the hypothesis that correct folding requires binding partners to provide the correct three‐dimensional context, and these are likely provided by other laminins, such as LAMB1 and seen in the structure of the αβγ complex in 5xau. To test this hypothesis, we predicted the structure of LAMA5 together with an expanded version of LAMB1 (residues 1487–1786) and the result is shown in Figures 6(c) and 6(d). As expected, the helices are now bundled together showing that the coiled coil motif provides sufficient complementarity to yield a much more reasonable arrangement of the helices in 3D space. The structure shown in Figure 6(d) is the same as in 6(c) but from a different angle. Figure 6(c) maintains the same orientation as Figures 6(a) and 6(b) to allow direct comparison, while Figure 6(d) is rotated to highlight the coiled coil nature visible in two triple‐helix coiled coils, the hallmark of coiled coil structures. To help differentiate between LAMA5 and LAMB1, and highlight the different individual helical regions involved, we used a yellow–orange gradient for LAMA5 and cyan–blue gradient for LAMB1. In both triple‐helix coils, LAMA5 provides two helices folded back on each other, while LAMB1 provides one. It is possible that a dimeric laminin structure might be possible, although only trimeric structures have been observed to date, with some evidence supporting the possibility of a βγ‐dimer being formed during trimer assembly [115, 116]. However, it is important to note that these studies have been carried out with fragments that lacked the contact sites involved in our predictions. Why are these predictions important? Highlighted in Figure 6 are the mutations associated with *LAMA5* and *LAMB1*. Ile1547Thr in *LAMB1* and Art2659Trp in *LAMA5* are located in the helical coiled coil regions. These mutations are expected to disrupt coiled coil propensity drastically, which has a very strict hydrophobic/hydrophilic amino acid pattern [117].

## 4. Discussion

The purpose of this study was to investigate five genetic hypotheses (Hs) related to CAS (H1 heterogeneity; H2 pleiotropy; H3 gene expression during the earliest brain development; H4 cerebellar locus of impairment; and H5 functional networks) by examining exome variants in three unrelated patients with CAS who have unaffected parents. As described below, we found evidence to support all five hypotheses.

Each of the three patients carried one or two *de* novo and several autosomal recessive and X‐linked exome variants. Despite the shared CAS diagnosis and phenotypic traits, no single shared exome variant was evident, nor were any implicated genes shared among the three patients. The most plausible genetic CAS influences were a *de novo LAMA5* variant for Patient 1, *de novo FOXN4* variant for Patient 2, and *de novo* variants in *USP17L2* and *LRRK2* and an autosomal recessive *LAMB1* variant for Patient 3. These findings are consistent with previous findings pointing to high levels of genotypic heterogeneity in CAS, consistent with H1 (heterogeneity).

In addition to examining phenotypes and exome variants, we investigated functional gene interactions and gene expression in the early developing brain. Phenotypically, the three probands, all male, shared slow babble development, late onset of first words, and motor speech deficits as evidenced in slow and inaccurate DDK performance, especially in the disyllabic condition. Additional shared traits were deficits in fine and gross motor coordination and signs of ASD. These shared traits are consistent with consistent with H2 (pleiotropy) and H4 (cerebellar locus of impairment).

Among the five priority genes, the highest gene expression rates in the BrainSpan data for the early developing cerebellum were seen for *LAMA5* and *LAMB1*. LAMA5 and LAMB1 are subunits in the well‐studied laminin‐511 complex which plays major roles in cell proliferation and migration throughout many tissues [118]. In the brain, laminin‐511 is known to protect neurons from cell death by inhibiting excitotoxicity and stabilizing dendritic spines and synapse [119]. The absence of laminin‐511 function is associated with behavioral defects in mice [119], and antibodies raised against it are toxic [120].

Each of the three participants carried variants in genes that are more highly expressed in the early developing cerebellum than in the average for the other brain regions, consistent with H4 (cerebellar locus of impairment), which is also relevant for ASD. In light of this, it is not surprising that all three patients in this study had received an ASD diagnosis at some point. These same findings also support the hypothesis that, to play a role in a neurodevelopmental disorder, such as CAS, contributing genes must be expressed in the earliest stages of neurodevelopment (H3, neurogenesis, and neural architecture). Our findings therefore extend current CAS genetics by adding an extracellular matrix and basement‐membrane–based mechanism to the predominantly chromatin‐regulatory pathways highlighted in recent gene‐discovery studies. Although lower‐priority genes were not examined in depth, their cerebellar‐skewed expression patterns suggest that additional contributors may influence the phenotypes observed, warranting future functional work.

PPI analysis of our CAS genes revealed a dense web of interactions with statistically significantly higher number of edges than predicted by chance, consistent with H5 (functional networks). These genes are involved in laminin complex formation, basement membrane and microtubule bundle, membrane–extracellular matrix interactions, and signal transduction pathways that regulate these processes. The only transcription factor in this network is *FOXN4*, the expression levels of which are controlled by many brain‐specific gene products, according to the Allen Brain Atlas. Several additional transcription factors had to be added to assist in connecting all nodes in the network. This highlights the role of transcription factors in regulating processes relevant for spoken communication, consistent with a recent review of chromatin‐based disorders that affect neurodevelopment as well as speech and language [46].

## 5. Clinical Implications

With a more advanced knowledge of the biological causes of CAS, we can move toward the development of proactive and personalized interventions, consistent with the tenets of ∗precision medicine. Conventional treatment of CAS is lengthy and costly and cannot begin until CAS is diagnosed, typically not until a child is 3 years or older, as is common practice currently. By this age, children have most likely missed extensive periods of speech practice in the form of babbling and are demonstrating marked frustration surrounding communication due to impaired ability to communicate basic wants and needs. Therapy that is deficit‐based requires unlearning faulty motor patterns, which adds extra burden to the process of acquiring intelligible speech. By developing proactive methods for the earliest possible interventions (i.e., babble therapy and parent education) for children at genetic risk for speech and language delays, we may support speech and language development from birth, leading to substantially improved outcomes and decreased cost of treatment. An example of this approach is Babble Boot Camp, designed for infants at predictable risk for severe speech and language disorders, here via a newborn diagnosis of CG. CG is an inborn error of metabolism that leads to severe disorders of expressive language and speech production, especially in the form of CAS, in 40%–80% of children with this condition. Babble Boot Camp is an intervention implemented via parent training, designed for children 2 months to 2 years. Treatment targets include increased vocalization rates, increased frequency and quality of babble, increasing receptive and expressive vocabulary, and expanding syntactic complexity. Because CG is diagnosed at birth and the risks for speech and language are known and predictable, infants with CG were selected for a clinical trial of Babble Boot Camp. Early pilot results were encouraging [121]. The first postintervention assessments of 13 children with CG who completed the program show no language delays in any of them and speech delays in only one [122, 123]. The intervention was rated as highly feasible, accessible, and beneficial by the parents of participating children [124]. Enhanced knowledge of other genetic triggers of CAS may pave the way to the earliest diagnosis via genotyping instead of waiting until phenotypes can be assessed, and to preventing or mitigating the emergence of full‐blown CAS via proactive interventions. Leveraging the known genotype–phenotype association toward a proactive approach and individualizing the intervention are two aspects of precision medicine translated into the realm of speech‐language pathology [76, 122, 123, 125].

## 6. Limitations and Future Directions

This study was restricted to exomic data, which means that variations outside of the coding regions were not investigated. Future studies of full genome sequences in trios with CAS may provide additional meaningful evidence toward biological causes of the disorder. The cerebellar locus of impairment was inferred based on phenotypic observations and the BrainSpan database; more direct observations via MRI studies should be conducted to validate the present findings. Future studies should investigate additional cases with CAS, both male and female, for greater insights into gene networks associated with the CAS phenotypes.

## Author Contributions

Caitlin Raaz: conceptualization, data curation, formal analysis, funding acquisition, and writing–first draft, review, and editing. Laurel Bruce: conceptualization, data curation, formal analysis, and writing–review and editing. Madhavi Ganapathiraju: formal analysis and writing–review and editing. Judith Klein‐Seetharaman: formal analysis, funding acquisition, and writing–review and editing. Li Liu: conceptualization and writing–review and editing. Valentin Dinu: conceptualization and writing–review and editing. Marjan Chapi: formal analysis and writing–review and editing. Eunhyo Kim: formal analysis and writing–review and editing. Yookyung Kim: formal analysis and writing–review and editing. Tiffanie White: formal analysis and writing–review and editing. Beate Peter: conceptualization, formal analysis, original draft, funding acquisition, and writing–first draft, review, and editing.

## Funding

This work was supported by NIDCD (5R03DC010886) to Beate Peter. Exome sequencing was provided by the University of Washington Center for Mendelian Genomics (UW‐CMG) and was funded by NHGRI (3UM1HG006493‐08S2) and (3U24HG008956‐04S1). Arizona State University New Faculty Startup Funds were awarded to Judith Klein‐Seetharaman and Beate Peter.

## Disclosure

A part of the individual findings regarding one of the three participants has been published in a dissertation that is available via Open Access: https://core.ac.uk/download/pdf/158457174.pdf. Partial findings regarding the other two participants individually have been published in a dissertation, also via Open Access: https://keep.lib.asu.edu/items/158859. The content is solely the responsibility of the authors and does not necessarily represent the official views of the National Institutes of Health.

## Ethics Statement

This work was completed with the approval of the Institutional Review Boards at the University of Washington and Arizona State University.

## Consent

Parents gave written permission for their minor children to participate in the study. Adults gave written consent for their own participation.

## Conflicts of Interest

The authors declare no conflicts of interest.

## General Statement

The adaptation of a table from Kaspi et al. [13]; https://doi.org/10.1038/s41380-022-01764-8) falls under the Creative Commons Attribution 4.0 International License (https://creativecommons.org/licenses/by/4.0/) that provides unrestricted use, distribution, modification, and reproduction in any medium.

## Endnotes

^1^Starred and underlined terms are listed and defined in Supporting File SF1.

## Supporting Information

Additional supporting information can be found online in the Supporting Information section.

## Supporting information


**Supporting Information 1** Supporting Table 1: Genes of interest for neurodevelopment, adapted from Kaspi et al. [13]; https://doi.org/10.1038/s41380-022-01764-8).

**Supporting Information 2** Supporting File 1: Glossary of technical terms.

## Data Availability

The DNA sequences are available through the controlled‐access database AnVIL (https://anvilproject.org/) under study accession ID phs003047.

## Appendix

BioGRID: https://thebiogrid.org/

BrainSpan Atlas of the Developing Human Brain: https://www.brainspan.org, https://www.brainspan.org/rnaseq/search/index.html

Cytoscape: https://cytoscape.org/

ClinVar: https://www.ncbi.nlm.nih.gov/clinvar/

gnomAD: https://gnomad.broadinstitute.org/

Human Protein Reference Database: https://ngdc.cncb.ac.cn/databasecommons/database/id/1383

Integrative Genomics Viewer: https://igv.org/

Panther: https://geneontology.org/docs/go-enrichment-analysis/

STRING: https://string-db.org

Variant Effect Predictor: https://useast.ensembl.org/info/docs/tools/vep/index.html

WikiPI: https://severus.dbmi.pitt.edu/wiki-pi/
