# Supplementary material for: Three Unrelated Children With Childhood Apraxia of Speech: Exome Sequencing and Functional Gene Analysis Imply a Role of Laminin‐511 in Early Neurodevelopment
Source: Case Rep Genet. 2026 Feb 13;2026:9927839. doi: 10.1155/crig/9927839 (PMC12905466; doi:10.1155/crig/9927839)
Supplement: Supplementary file 2 — Supporting Information 2 Supporting File 1: Glossary of technical terms. [file CRIG-2026-9927839-s002.docx]

**Appendix: Glossary**

Note: Terms that refer to other terms in the glossary are underlined and starred.

16p11.2 band (and other bands): Chromosomes are divided into specific regions (“bands”) to make it easier to describe the locations of certain genes. The notation scheme for these bands is as follows: Chromosome number, p or q arm, numbered band starting at the centromere where the p and q arms are joined, period symbol, subregion.

Autosomal recessive: A condition resulting from two genetic variants, inherited from both parents.

Chromatin organization: Chromatins are structural elements that support the organization of long strands of DNA into systematic coiled patterns.

Copy-number variation (CNV): A part of a chromosome was deleted or duplicated, so that the individual carries one or three copies of that part of the chromosome instead of the expected two copies.

*De novo*: A genetic change that was not inherited from a parent; rather, it arose sporadically.

DNA binding protein: A protein that attaches itself to a specific region of DNA, for instance to regulate the function of a gene in this region.

Exome: The entire sum of an individual’s protein-coding DNA, which amounts to approximately 1% to 2% of the individual’s entire DNA.

G proteins: Proteins that communicate signals transmitted from outside a cell into the inside of the cell where they elicit a response.

Genotype: An individual’s DNA profile at a given gene position.

Genotyping: Determining an individual’s DNA profile at chromosomal regions of interest.

GO term enrichment analysis: The Gene Ontology (GO) is a hierarchy of terms related to three aspects of biological knowledge about a gene: (1) molecular function, (2) cellular component and (3) biological process. Looking for statistically significant overrepresentation of terms in a given set of genes is frequently used to associate that group with a biological function. This can be useful for example in the analysis of *-omics data because there are typically multiple genes involved so that we can look for commonalities amongst those genes even if they are not closely related in sequence.

Heterogeneity, heterogeneous: Individuals with a similar condition carry different genetic mechanisms that caused their condition.

Hemizygous: A variant on the X chromosome of a male individual is called hemizygous because there is no corresponding position on a second X chromosome because males carry an X and a Y chromosome.

Heterozygous, heterozygosity: Humans carry two copies of 22 chromosomes. The DNA sequence in a given region of these copies may be identical; e.g., an individual could carry an A at a given DNA sequence position on one chromosome and a G at the same position on the other chromosome, a scenario that is called heterozygous or heterozygosity.

Homozygous: Humans carry two copies of 22 chromosomes. The DNA sequence in a given region of these copies may be identical; e.g., an individual could carry an A at the same DNA sequence position in each of the two chromosomes, a scenario that is called homozygous.

Human accelerated region (HAR): A set of 49 DNA regions that are highly conserved across vertebrates but notably different in humans. It is thought that these regions evolved rapidly, contributing to specifically human traits.

Interactome: The entirety of interactions between biomolecules, typically between proteins; i.e., the network of *protein-protein interactions (PPIs) within an organism, or across organisms, e.g. in host-pathogen interactions. In an interactome, the edges connecting nodes can for example represent physical interactions or genetic or functional linkages, co-occurrence in a publication, or co-expression under a given condition.

Loss-of-function variant: A change in the DNA sequence of a gene that interferes with the gene’s functional properties.

Minor allele frequency: The frequency with which a *variant occurs in the population.

Nucleotide: Building blocks of DNA. There are four nucleotides, adenine, cytosine, guanine, and thymine. Their sequential arrangement in a gene determines the function of the gene.

-Omics: A term that collectively refers to those disciplines in the biological sciences that end in the suffix “omics” such as genomics, proteomics, and metabolomics. Within these disciplines, the entirety of data is sudied, e.g., genomics refers to the study of all genes whereas genetics refers to the study of specific genes.

Phenotype: Observable characteristics.

Pleiotropic: Referring to two or more traits that may appear unrelated but are caused by the same genetic variation.

Precision medicine: An emerging approach to managing diseases by taking into consideration an individual’s risk profile based on genetic makeup, environmental influences, and lifestyle factors. The goal is to improve outcomes by matching interventions to each patient’s risk profile and by taking preventive measures in the presence of known risks.

Probability of loss-of-function intolerance (pLI): A measure of how many protein-altering variants are observed in living organisms as a function of how many would be expected by chance, given a gene’s length. If significantly fewer variants are observed than expected, this implies that the gene is intolerant of protein-altering variants.

Protein-protein interaction (PPI): Highly specific biochemical events between two or more proteins. PPIs influence biological processes such as interactions between cells, metabolic events, and cell growth and development.

Single nucleotide variant (SNV): A single DNA letter was changed from the most commonly occurring version to a more unusual one.

Terminus (N-terminus / C-terminus: Proteins are made of peptide chains built from amino acids. Each amino acid has an amino group (–NH₂) and a carboxyl group (–COOH). As a result, every peptide chain has two ends: it starts with an amino group (the N-terminus) and finishes with a carboxyl group (the C-terminus).

Transcriptional regulation: Biological processes that act as “on” or “off” switches determining when a gene is activated.

Translocation: A part of a chromosome broke off and became attached to a different chromosome.

Transition missense *variant: A missense variant is a change in the DNA sequence from one “letter” to another one. If the two letters are of the same type, e.g., an A changes to a G (both are classified as purines), the variant is called a transition missense variant.

Variant: A change in an individual’s DNA sequence that differs from that found in the reference population. Formerly referred to as mutation.

Whole-exome sequencing: Creating the DNA sequence of an individual by selecting only those parts of the DNA that contain the protein-coding parts of genes.

X-linked *de novo*: A variant on the X chromosome that was not inherited from a parent.

X-linked recessive: For an individual to show an x-linked recessive genes, females must carry two identical copies of the causal *variant, whereas affected males only have one copy of the variant.
